# Supplementary material for: Transcriptomic Insights into Mechanisms of Early Seed Maturation in the Garden Pea (Pisum sativum L.)
Source: Cells. 2020 Mar 23;9(3):779. doi: 10.3390/cells9030779 (PMC7140803; doi:10.3390/cells9030779)
Supplement: Supplementary file 1 [file cells-09-00779-s001.zip › Supplementary materials/FileS3.zip!/FileS2/Hormones - Results/Summary Sheets - Modules/Group Overexpression Spots/Report.pdf]

Group Overexpression Spots

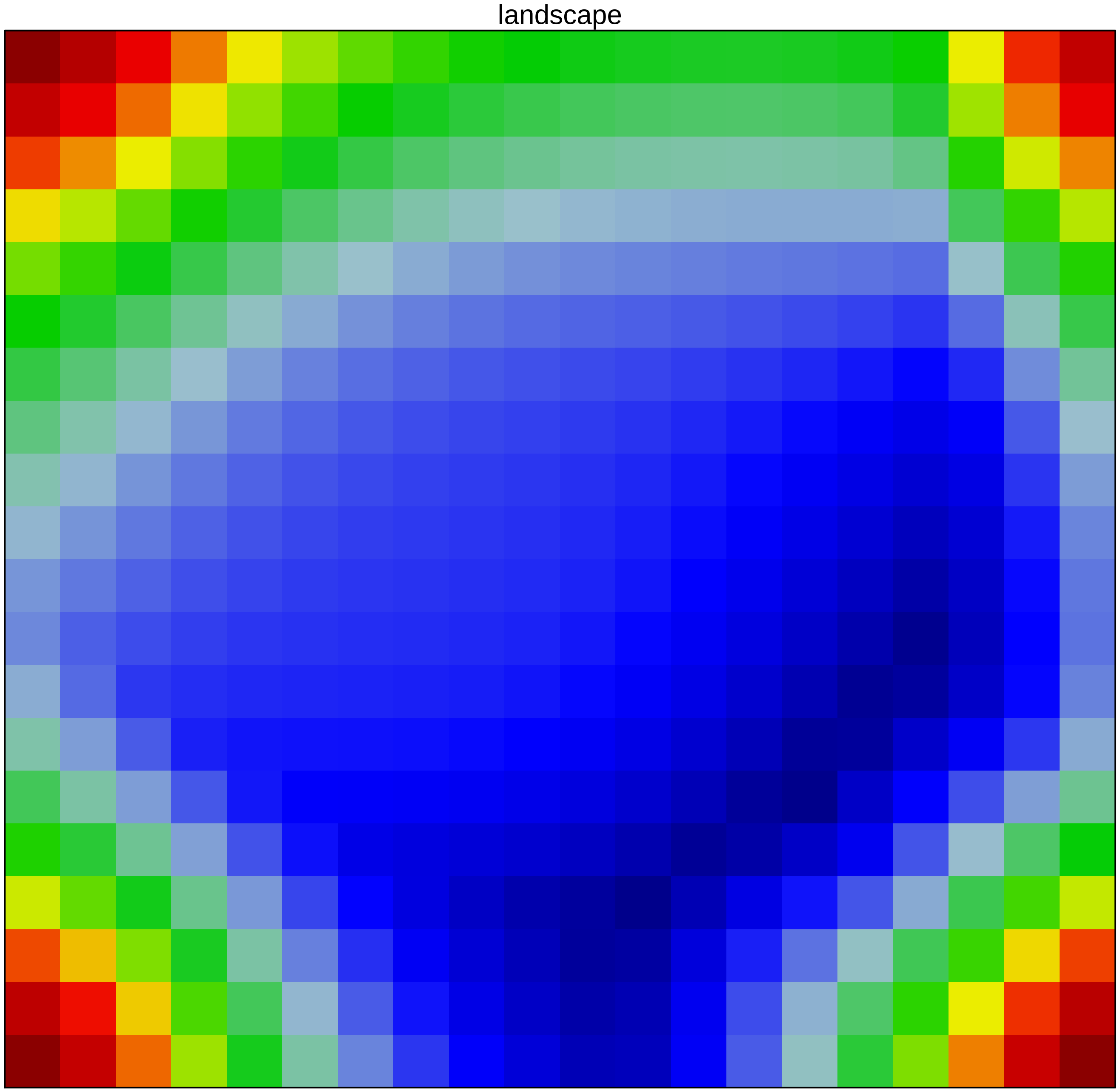

Group Overexpression Spots

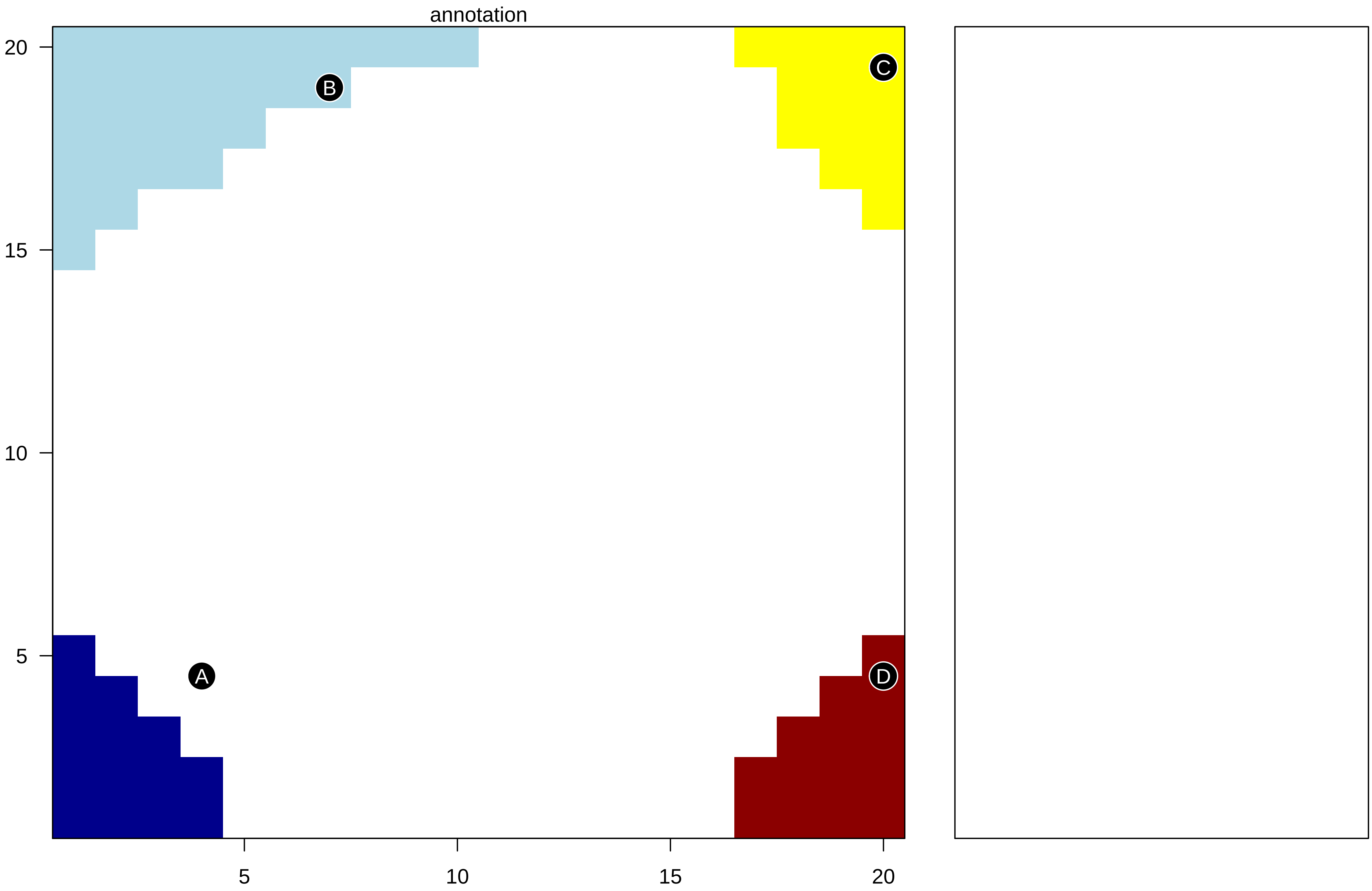

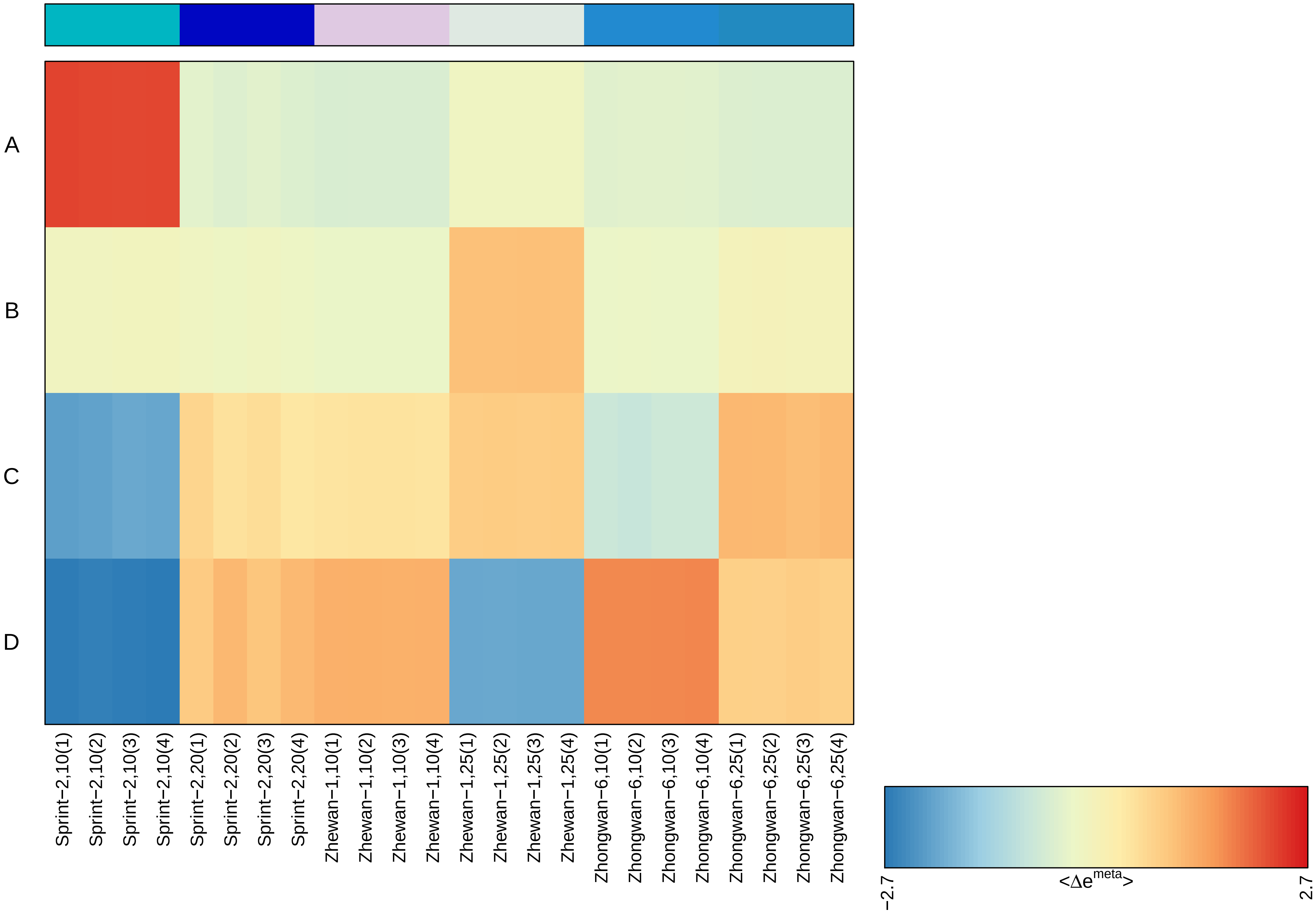

A
B
C
D
Sprint−2,10(1)
Sprint−2,10(2)
Sprint−2,10(3)
Sprint−2,10(4)
Sprint−2,20(1)
Sprint−2,20(2)
Sprint−2,20(3)
Sprint−2,20(4)
Zhewan−1,10(1)
Zhewan−1,10(2)
Zhewan−1,10(3)
Zhewan−1,10(4)
Zhewan−1,25(1)
Zhewan−1,25(2)
Zhewan−1,25(3)
Zhewan−1,25(4)
Zhongwan−6,10(1)
Zhongwan−6,10(2)
Zhongwan−6,10(3)
Zhongwan−6,10(4)
Zhongwan−6,25(1)
Zhongwan−6,25(2)
Zhongwan−6,25(3)
Zhongwan−6,25(4)
−2.7
<Δe^meta>
2.7

Group Overexpression Sp[illegible]

Spot Summary: A

metagenes = 14
genes = 21

<r> metagenes = 1
<r> genes = 0.82
beta: r2= 1.73 / log p= −Inf

samples with spot = 4 (16.7 %)
Sprint−2(10) : 4 (100 %)

Overview Map

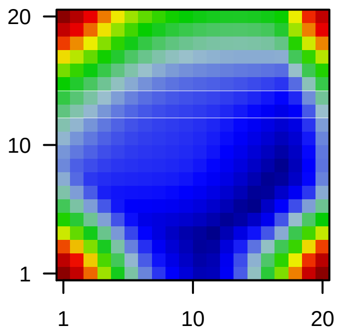

Spot

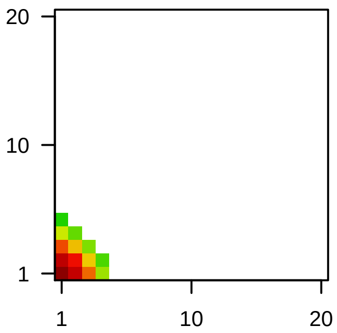

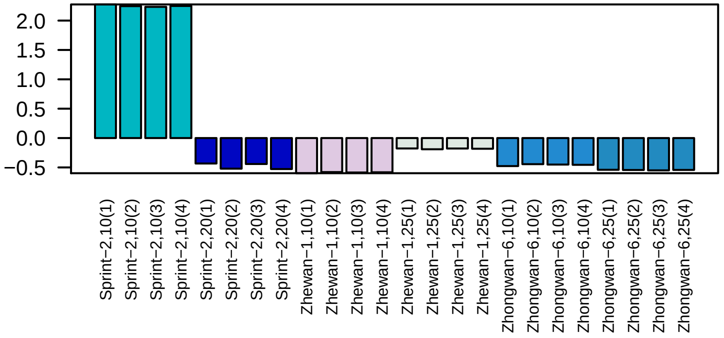

Spot Genelist

Rank	ID	max e	min e	r	Symbol	Description
1	TRINITY_DN1	15.48	−3.34	0.91		
2	TRINITY_DN3	15.03	−3.06	0.97		
3	TRINITY_DN4	4.64	−1.14	1		
4	TRINITY_DN8	3.6	−0.71	0.99		
5	TRINITY_DN4	3.56	−0.75	0.98		
6	TRINITY_DN8	3.06	−0.71	0.98		
7	TRINITY_DN6	2.31	−0.98	0.36		
8	TRINITY_DN3	1.81	−0.35	0.89		
9	TRINITY_DN3	1.66	−0.44	0.96		
10	TRINITY_DN1	1.51	−0.8	0.82		
11	TRINITY_DN5	1.42	−0.47	0.86		
12	TRINITY_DN1	1	−0.39	0.83		
13	TRINITY_DN2	0.93	−0.39	0.97		
14	TRINITY_DN1	0.85	−0.17	0.98		
15	TRINITY_DN2	0.69	−0.19	0.97		
16	TRINITY_DN8	0.62	−0.27	0.72		
17	TRINITY_DN1	0.54	−0.21	0.96		
18	TRINITY_DN6	0.54	−0.13	0.96		
19	TRINITY_DN6	0.48	−0.11	0.99		
20	comp22899_c	0.4	−0.19	0.91		

Group Overexpression Spo

Spot Summary: B

metagenes = 29
genes = 24

<r> metagenes = 0.91
<r> genes = 0.74
beta: r2= 0.44 / log p= −3.35

samples with spot = 0 (0 %)

Overview Map

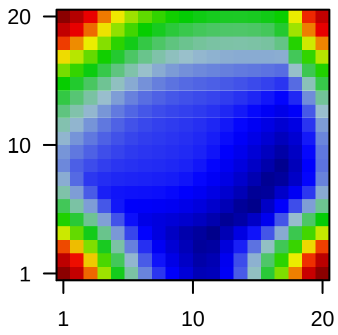

Spot

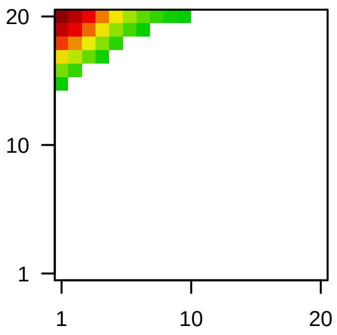

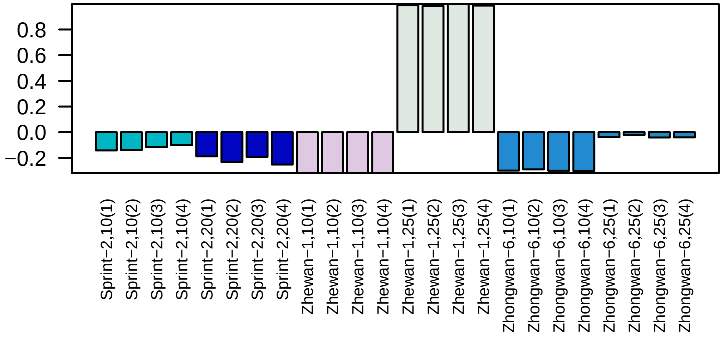

Spot Genelist

Rank	ID	max e	min e	r	Symbol	Description
1	TRINITY_DN3	15.54	−3.43	0.98		
2	TRINITY_DN1	1.68	−0.51	0.99		
3	TRINITY_DN1	0.79	−0.37	0.92		
4	TRINITY_DN5	0.75	−0.46	0.69		
5	TRINITY_DN5	0.74	−0.39	0.91		
6	TRINITY_DN4	0.59	−0.48	0.82		
7	TRINITY_DN1	0.52	−0.35	0.76		
8	TRINITY_DN5	0.47	−0.16	0.97		
9	TRINITY_DN1	0.4	−0.22	0.86		
10	TRINITY_DN8	0.37	−0.25	0.62		
11	TRINITY_DN3	0.29	−0.14	0.76		
12	TRINITY_DN6	0.28	−0.1	0.95		
13	TRINITY_DN3	0.26	−0.26	0.43		
14	TRINITY_DN1	0.22	−0.06	0.99		
15	TRINITY_DN4	0.22	−0.09	0.91		
16	TRINITY_DN5	0.21	−0.28	0.63		
17	TRINITY_DN1	0.2	−0.07	0.98		
18	TRINITY_DN3	0.2	−0.1	0.89		
19	TRINITY_DN1	0.2	−0.06	0.98		
20	TRINITY_DN1	0.19	−0.11	0.93		

Group Overexpression Sp[illegible]

Spot Summary: C

metagenes = 13
genes = 8

<r> metagenes = 0.99
<r> genes = 0.55
beta: r2= 2.54 / log p= −Inf

samples with spot = 0 (0 %)

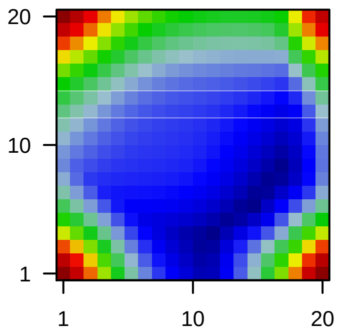

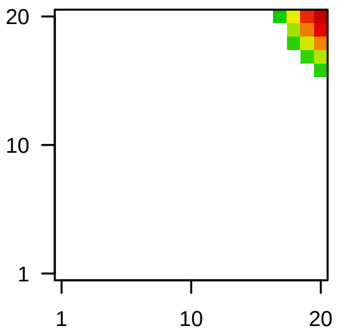

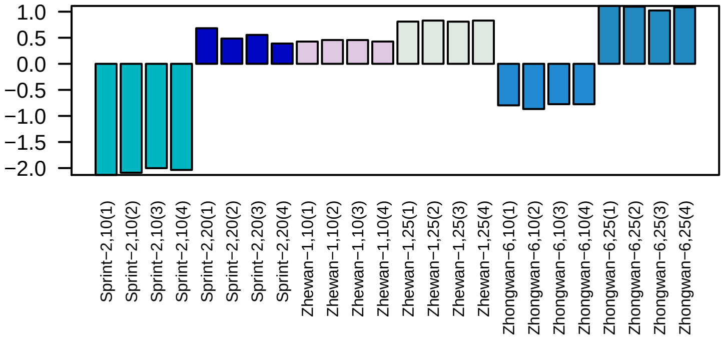

Spot Genelist

Rank	ID	max e	min e	r	Symbol	Description
1	TRINITY_DN1	3.01	−8.1	0.98		
2	TRINITY_DN3	2.24	−1.85	0.77		
3	TRINITY_DN4	1.68	−0.51	0.56		
4	TRINITY_DN6	1.58	−2.5	0.89		
5	TRINITY_DN2	1.45	−0.95	0.56		
6	TRINITY_DN7	1.1	−1.67	0.67		
7	TRINITY_DN6	0.64	−0.76	0.85		
8	TRINITY_DN3	0.57	−0.79	0.58		

Group Overexpression Spo

Spot Summary: D

metagenes = 14
genes = 10

<r> metagenes = 1
<r> genes = 0.48
beta: r2= 4.67 / log p= −Inf

samples with spot = 8 (33.3 %)
Zhewan−1(10) : 4 (100 %)
Zhongwan−6(10) : 4 (100 %)

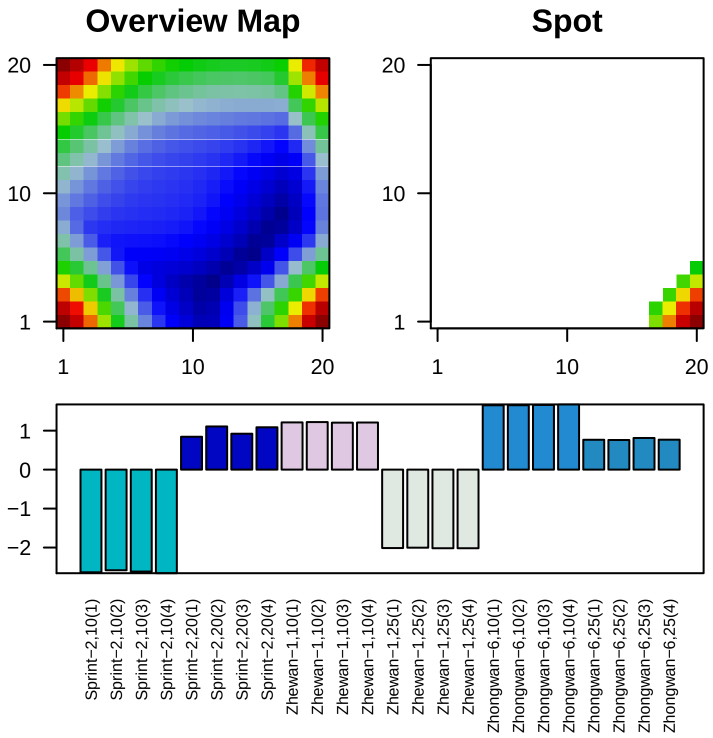

Spot Genelist

Rank	ID	max e	min e	r	Symbol	Description
1	TRINITY_DN1	9.22	−1.86	0.58		
2	TRINITY_DN3	8.41	−2.58	0.38		
3	TRINITY_DN1	5.71	−13.31	0.99		
4	TRINITY_DN5	3.72	−1.76	0.48		
5	TRINITY_DN2	2.73	−3.16	0.83		
6	TRINITY_DN4	1.96	−0.45	0.49		
7	TRINITY_DN2	1.83	−1.59	0.53		
8	TRINITY_DN3	1.75	−1.32	0.83		
9	TRINITY_DN6	1.68	−1.13	0.6		
10	TRINITY_DN3	0.64	−0.6	0.94		
